# The complete chloroplast genome sequence of *Populus davidiana* (Salicaceae)

**DOI:** 10.1080/23802359.2020.1768937

**Published:** 2020-05-28

**Authors:** Haiyan Yu, Li Zhang, Jin Zang, Liandong Xiao, Jiancheng Luo

**Affiliations:** a School of Biological and Chemical Engineering, Nanyang Institute of Technology, Nanyang, PR, China; b Henan Key Laboratory of Industrial Microbial Resources and Fermentation Technology, Nanyang, PR, China

**Keywords:** *P. davidiana*, chloroplast genome, phylogenetic analysis, genetic information

## Abstract

The complete chloroplast genome sequence of *Populus davidiana* was characterized from Illumina pair-end sequencing. The chloroplast genome of *P. davidiana* was 156,868 bp in length, containing a large single-copy region (LSC) of 84,976 bp, a small single-copy region (SSC) of 16,606 bp, and two inverted repeat (IR) regions of 27,643 bp. The overall GC content is 30.70%, while the correponding values of the LSC, SSC, and IR regions are 64.6%, 69.2%, and 60.1%, respectively. The genome contains 131 complete genes, including 86 protein-coding genes (62 protein-coding gene species), 37 tRNA genes (29 tRNA species) and 8 rRNA genes (4 rRNA species). The Neighbour-joining phylogenetic analysis showed that *P. davidiana* and *Populus rotundifolia* clustered together as sisters to other *Populus* species.

*Populus davidiana* Dode (Salicaceae) is among the most geographically widespread (across latitudes) and ecologically important tree species in China, which has persisted largely in an undomesticated state that is highly resistant to different environmental stresses (Hou et al. 2018). *Populus davidiana* has high ecological and economic value with high levels of intraspecific genetic diversity. A recent study of *P. davidiana* based on 6 nuclear and 3 chloroplast loci suggests that three distinct groups, from the Northeast, central and Southwest China exist and that a refugium might have existed in Northeast China during the last glacial maximum (LGM) (Hou et al. 2018).

*Populus davidiana* has wide geographic distribution, high intraspecific polymorphism, adaptability to different environments, combined with a relatively small genome size. Consequently, *P. davidiana* represents an excellent model for understanding how different evolutionary forces have sculpted the variation patterns in the genome during the process of population differentiation and ecological speciation (Neale and Antoine 2011). Moreover, we can develop conservation strategies easily when we understand the genetic information of *P. davidiana*. In the present research, we constructed the whole chloroplast genome of *P. davidiana* and understood many genome variation information about the species, which will provide beneficial help for population genetics studies of *P. davidiana.*

The fresh leaves of *P. davidiana* were collected from Changchun city (43°48′N, 125°19′E). Fresh leaves were silica-dried and taken to the laboratory until DNA extraction. The voucher specimen (ZGSY001) was laid in the Herbarium of Nanyang Institute of Technology and the extracted DNA was stored at –80 °C in the refrigerator of the Key Laboratory of School of Biological and Chemical Engineering. We extracted total genomic DNA from 25 mg silica-gel-dried leaf using a modified CTAB method (Doyle 1987). The whole-genome sequencing was then conducted by Biodata Biotechnologies Inc. (Hefei, China) with Illumina Hiseq platform. The Illumina HiSeq 2000 platform (Illumina, San Diego, CA) was used to perform the genome sequence. We used the software MITObim 1.8 (Hahn et al. 2013) and metaSPAdes (Nurk et al. 2017) to assemble chloroplast genomes. We used *P. tremula* (GenBank: NC_027425) as a reference genome. We annotated the chloroplast genome with the software DOGMA (Wyman et al. 2004), and then corrected the results using Geneious 8.0.2 (Campos et al. 2016) and Sequin 15.50 (http://www.ncbi.nlm.nih.gov/Sequin/).

The complete chloroplast genome of *P. davidiana* (GenBank accession number MG262347) was characterized using Illumina pair-end sequencing. The chloroplast genome of *P. davidiana* was 156,868 bp in length, containing a large single-copy region (LSC) of 84,976 bp, a small single-copy region (SSC) of 16,606 bp, and two inverted repeat (IR) regions of 27,643 bp. The overall GC content is 30.70%, while the corresponding values of the LSC, SSC, and IR regions are 64.6%, 69.2%, and 60.1%, respectively. The genome contains 131 complete genes, including 86 protein-coding genes (62 protein-coding gene species), 37 tRNA genes (29 tRNA species), and 8 rRNA genes (4 rRNA species).

We used the complete chloroplast genomes sequence of *P. tremula* and 12 other related species of *Populus* and Salix interior as an outgroup to construct the phylogenetic tree. The 14 chloroplast genome sequences were aligned with MAFFT (Katoh and Standley, 2013), and then the Neighbour-joining tree was constructed by MEGA 7.0 (Kumar et al. 2016). The results confirmed that *P. davidiana* was clustered with *P. rotundifolia* (Figure 1).

**Figure 1.**
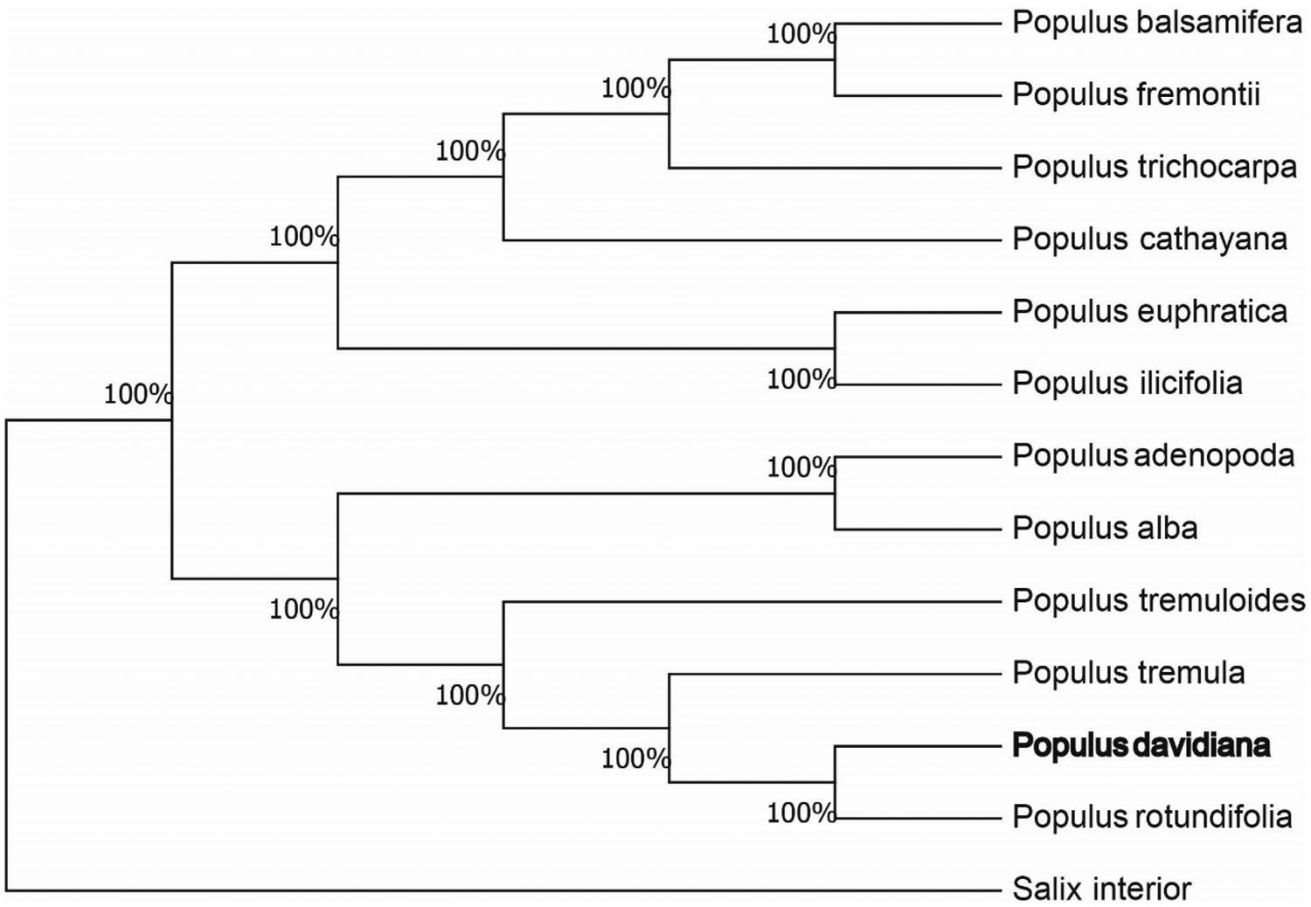
Neighbour-joining (NJ) analysis of *P. davidiana* and other related species based on the complete chloroplast genome sequence. Genbank accession numbers: *P. tremula* (KP861984), *P. koreana* (MN864049), *P. yunnanensis* (KP729176), *P. euphratica* (KJ624919), *P. adenopoda* (NC032368), *P. rotundifolia* (KX425853), *P. cathayana* (KP929175), *P. balsamifera* (KJ664927), *P. ilicifolia* (NC031371), *P. trichocarpa* (EF489041), *P. fremontii* (KJ664926), *P. tremuloides* (MN561844) and *Salix interior* (NC024681).

## Data Availability

The GenBank accession number for the cp genome sequence of *P. davidiana* is MG262347 and the DOI is https://www.ncbi.nlm.nih.gov/nuccore/MG262347.1.
